# Maximal exercise in obese patients with COPD: the role of fat free mass

**DOI:** 10.1186/1471-2466-14-96

**Published:** 2014-05-30

**Authors:** Marina Aiello, Elisabetta Teopompi, Panagiota Tzani, Sara Ramponi, Maria Rosaria Gioia, Emilio Marangio, Alfredo Chetta

**Affiliations:** 1 Dept of Clinical & Experimental Medicine, Respiratory Disease and Lung Function Unit, University of Parma, Strada dell’Università 12, Parma 43100, Italy

**Keywords:** Fat-free mass, Dynamic hyperinflation, Exercise, Obesity, COPD

## Abstract

**Background:**

Obese patients (OB) with COPD may better tolerate exercise as compared to normal weight (NW) COPD patients, even if the reason for this is not yet fully understood. We investigated the interactions between obesity, lung hyperinflation, fat-free mass (FFM) and exercise capacity in COPD.

**Methods:**

Forty-four patients (16 females; age 65 ± 8 yrs) were assessed by resting lung function and body composition and exercised on a cycle-ergometer to exhaustion.

**Results:**

Twenty-two OB and 22 NW patients did not differ in age, gender and airflow obstruction degree, but in FFM (p < 0.05). OB had significantly higher values in inspiratory capacity/total lung capacity ratio (IC/TLC) at rest (p < 0.01), but not at peak of exercise and showed significantly higher values in peak workload (p < 0.05) and in peak oxygen uptake (VO_2_), when expressed as absolute value (p < 0.05), but not when corrected by FFM. OB compared to NW experienced lower leg fatigue (p < 0.05), but similar dyspnea on exertion. In all patients, the regression equation by stepwise multiple regression analysis for peak workload and VO_2_, as dependent variables included both FFM and IC/TLC at rest, as independent variables (r^2^ = 0.43 and 0.37, respectively).

**Conclusions:**

OB with COPD, as compared to NW patients matched for age, gender and airflow obstruction, had greater FFM and less resting lung hyperinflation and showed greater maximal exercise capacity. Pulmonary and non-pulmonary factors may explain the preservation of exercise tolerance in patients with COPD associated with obesity.

## Background

Obesity, an independent risk factor for reduced survival [1], is a frequent co-morbidity in patients with chronic obstructive pulmonary disease (COPD) [2]. Surprisingly, epidemiological studies have shown that overweight or mild-to-moderate obese patients with COPD have a survival advantage compared to underweight patients [3,4], even if the reason for this is not yet clear [5]. It is also of note that, despite the increase in metabolic and ventilatory requirements, obesity also has the potential for significant beneficial effects on exercise tolerance in COPD [6].

The impact of obesity on exercise capacity is not completely understood. Previous studies have shown that the combined mechanical effects of obesity and COPD may reduce operating lung volumes at rest and during exercise with favourable influences on breathlessness perception and peak oxygen uptake [7,8]. On the other hand, in COPD patients the decreased physical activity does not seem to depend merely on resting lung function and other factors are considered important in contributing to stagnation. Fat free mass (FFM) depletion commonly occurs in COPD patients and results from several factors, such as systemic inflammatory mediators, disuse atrophy, poor nutrition and oral corticosteroid medication [9]. The reduction in FFM may further significantly contribute to the impairment of exercise capacity in these patients. Previous studies have reported a significant correlation between FFM and maximal [10] and sub-maximal [11] exercise capacity in COPD patients. Furthermore, the depletion of lean body mass can reduce exercise capacity independently from ventilatory constraints during exertion [12].

Interestingly, in obese subjects, FFM may be increased [13,14] as adaptation to the sustained loading effect from excessive adipose tissue. Therefore, we hypothesized that even in obese patients with COPD, an increase in FFM may occur with favourable effects on exercise capacity. In order to verify this hypothesis, we measured FFM in a group of obese patients with diagnosis of COPD and in a control group of normal weight COPD patients matched for age, gender and airflow obstruction. Then, we compared their cardiovascular and ventilatory response to incremental maximal exercise testing.

## Methods

### Patients

We consecutively enrolled patients affected by COPD, who were admitted to a pulmonary rehabilitation program. All patients underwent routine blood and urine analysis as an initial screening prior to the rehabilitation program. COPD was diagnosed according to the GOLD criteria [15] and patients with moderate to severe airflow obstruction, i.e., Forced Expiratory Volume in 1^st^ Second/Vital Capacity ratio (FEV_1_/VC) < 70% and FEV_1_ ≤ 80% of predicted value, were included. Eligibility criteria were: 1) stable clinical condition (exacerbation free for at least 4 weeks); 2) no oxygen therapy; 3) absence of any comorbidity affecting exercise performance (anaemia, neuromuscular disorders, chronic cardiac failure, malignancies); 4) ability to perform a symptom-limited cycle ergometry cardio pulmonary test (CPET) with a peak of respiratory exchange ratio (RER) ≥ 1.05 in order to exclude poor motivation; 5) CPET stopped for muscle fatigue and/or dyspnoea.

All the procedures and their risks were explained to the patients, who gave their written informed consent to enter the study. The protocol was approved by the ethical committee of the University Hospital of Parma. All participants' data were analysed and reported anonymously.

### Lung function

Pulmonary function tests were performed according to international recommendations [16,17]. A flow-sensing spirometer and a body plethysmograph connected to a computer for data analysis (Vmax 22 and 6200, Sensor Medics, Yorba Linda, U.S.A.) were used for the measurements. Vital Capacity (VC), Forced Expiratory Volume at 1^st^ Second (FEV_1_), Forced Expiratory Flow measured at 50% of Forced Vital Capacity (FVC) (FEF_50_ in L/s) and Forced Inspiratory Flow measured at 50% of FVC (FIF_50_ in L/s) were recorded. FEV_1_/VC and FEF_50_/FIF_50_ ratios were taken as indices of airway obstruction and airway collapsibility, respectively.

Functional Residual Capacity (FRC) was measured by body plethysmography with the subject panting against a closed shutter at a frequency slightly lower than 1 Hz whilst supporting their cheeks with their hands. Total Lung Capacity (TLC) was obtained as the sum of FRC and linked Inspiratory Capacity (IC). Residual Volume (RV) was obtained by subtracting VC from TLC. IC/TLC ratio was taken as an index of hyperinflation of the lung. At least three measurements were taken for each spirometry and lung volume variable to ensure reproducibility and the highest value obtained was used in subsequent calculations. The flow-sensor was calibrated before each test using a three-litre syringe.

Lung diffusion capacity for carbon monoxide (TLco) was measured by the single breath method using a mixture of carbon monoxide and methane; this measurement was done at least in duplicate. TLC, VC, IC, FEV_1_ and TLco were expressed as a percentage of the predicted values, which were obtained from regression equations by Quanjer et al. [18] and Cotes et al. [19].

### Cardiopulmonary exercise test

CPET was performed prior to the rehabilitation program according to a standardized procedure [20]. After calibrating the oxygen and carbon dioxide analyzers and flow mass sensor, patients were asked to sit on an electromagnetically braked cycle ergometer (Corival PB, Lobe Bv, Groningen, The Netherlands) and the saddle was adjusted properly to avoid maximal extension of the knee. The exercise protocol involved an initial 3 minutes of rest, followed by unloaded cycling for another 3 minutes with an increment every minute of 5 to 15 W according to the patient’s anthropometry and degree of functional impairment, in order to achieve an exercise time between 8–12 min. Patients were asked to maintain a pedalling frequency of 60 rpm indicated by a digital display placed on the monitor of the ergometer. Breath-by-breath oxygen uptake (VO_2_ in L/min), carbon dioxide production (VCO_2_ in L/min), tidal volume (V_T_ in L) and minute ventilation (VE in L/min) were recorded during the test (CPX/D; Med Graphics, St Paul, MN, U.S.A.). Patients were continuously monitored with a 12-lead electrocardiogram (Welch Allyn CardioPerfect, Delft, the Nederlands) and a pulse oximeter (Pulse Oximeter 8600, Nonin Medical Inc, MPLS, Mn U.S.A.). Blood pressure was measured at 2 minute intervals. Stopping criteria consisted of symptoms such as unsustainable dyspnoea or leg fatigue, chest pain, ECG significant ST-segment depression, a drop in systolic blood pressure or oxygen saturation (SpO_2_) ≤ 84%.

Peak workload and peak VO_2_ were recorded as the mean value of watts and VO_2_ during the last 20 seconds of the test. Peak VO_2_ was expressed as absolute value in mL/min. Anaerobic threshold (AT) was non-invasively determined by both V-slope and ventilatory equivalents methods (“dual method approach”), as the respiratory exchange ratio approximated 1.0 [20], and was expressed as absolute value in mL/min. Oxygen pulse (O_2_Pulse, in mL/bpm) was calculated by dividing instantaneous oxygen uptake by heart rate [20] and was recorded at rest and at peak of exercise.

Changes in operational lung volumes were assessed every two minutes during exercise and at peak of exercise, taking the IC measured at rest as the baseline. After a full explanation of the procedure to each patient, satisfactory technique and reproducibility of IC manoeuvres were established during an initial practice session at rest. Assuming that TLC remains constant during exercise in COPD [21], changes in IC reflect changes in end-expiratory lung volume (EELV). The end-tidal pressure of CO_2_ (PETCO_2_, in mm Hg) was measured as the mean of PETCO_2_ during the 3-minute rest period and during the last 20 seconds of the test and the difference between PETCO_2_ peak and PETCO_2_ rest (PETCO_2_ peak-rest) was recorded.

### Dyspnoea and muscle fatigue

Daily living activity-related dyspnoea was evaluated with the Italian version of the five-point MRC scale modified by the ATS [22]. At the end of CPET patients were asked to name the predominant symptom limiting exercise. Dyspnoea and muscle fatigue induced by CPET were also measured at the end of incremental exercise by a visual analogue scale (VAS). The VAS scale consisted of a 100-mm horizontal line with the word “*none*” placed at the left end of the scale and the word “*very severe*” placed at the right end of the scale. The VAS scored from 0 to 100, but the subjects were unaware of the numbers. Dyspnoea and muscle fatigue ratings were then divided by the maximal workload (VAS_dys_ and VAS_fat_, respectively in mm/watts) [23].

### Body composition

Body height and weight were measured anthropometrically and body mass index (BMI, kg/m^2^) was calculated in all patients. Body composition was assessed by a bioelectrical impedance analysis (BIA) method, which is based on the conductance of an electrical sinusoidal alternating current through body fluids. BIA measures the impedance or resistance to the signal as it travels through the water that is found in muscle and fat. Foot-to-foot BIA was measured using a SC-331S Body Composition analyzer (TANITA CO., Tokyo, Japan). Patients were measured in a standing position with bare feet on the analyzer footpads. The algorithms used to estimate lean body mass from impedance are those given by Segal et al. [24]. Fat mass (FM) and FFM were measured in all patients. FFM was also standardized for height similar to BMI: FFM index (FFMI: FFM/height^2^, kg/m^2^).

### Statistical analysis

This is a cross-sectional pilot study. Due to the explorative nature of the study no formal sample size calculation was performed. Data are reported as mean ± standard deviation (SD), unless otherwise specified. The distribution of variables was assessed by means of the Kolmogorov-Smirnov Goodness-of-Fit test. Relationships between variables were assessed by the Pearson’s correlation coefficient (*r*) and linear regression analysis. Comparisons between variables were determined by unpaired *t* test and χ^2^ test, when appropriate. Stepwise multiple regression analysis was used to determine the best predictor variables for peak workload and VO_2_ as dependent variables. Percentage of total variance in the dependent variable, accounted for by the predictor variables, is expressed as the adjusted square of the multiple correlation coefficient (*r*^2^).

According to the BMI value [25], population sample was divided into normal weight (NW, BMI 18.5-24.9 kg/m^2^) and obese (OB, BMI ≥ 30 kg/m^2^) patients. According to IC/TLC, patients of both groups were also divided in two categories: patients with IC/TLC ≤ 0.25 or > 0.25 both at rest and at peak of exercise. In COPD a IC/TLC ratio > 0.25 at rest is an established favourable prognostic indicator [26] and at peak is associated to less negative cardiovascular effects [27].

A *p* value of less than 0.05 was taken as significant.

## Results

Sixty consecutive stable COPD patients (19 females), aged between 42 and 75 years were screened. All of them were ex-smokers. Sixteen patients were excluded because of their BMI (<19, ≥ 25 or < 30 kg/m^2^). Twenty-two NW patients (BMI range: 19.0–24.9 kg/m^2^) and 22 OB patients with COPD (BMI range: 30.1–44.8 kg/m^2^) were studied. Twenty-nine (8 females) out of 44 patients (66%) suffered from controlled arterial hypertension and were taking diuretics (38%), Ca-antagonists (34%), beta-blockers (34%), and ACE-inhibitors (28%). The prevalence of arterial hypertension was not different between NW and OB patients (64% vs 68%). Additionally, the percentage of patients on beta-blockers was not different.

**Figure 1:**
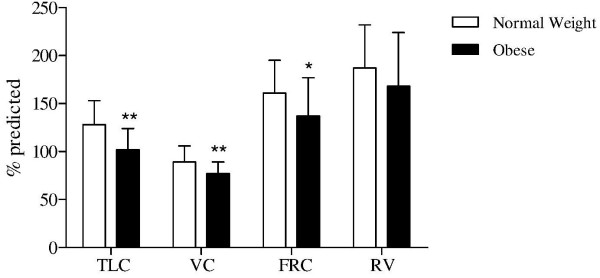
**Mean and standard deviation values of TLC, VC, FRC and RV in 22 normal weight and in 22 obese patients with COPD.** *p < 0.05, **p < 0.01.

Demographic and clinical characteristics of the 44 patients included in the study are shown in Table 1. The two groups of patients differed in FM, FFM and FFMI values, but not in age, gender and airflow obstruction and airway collapsibility degree. OB patients showed lower values in TLC, VC and FRC when compared to NW patients (Figure 1). In all patients, BMI was negatively related to TLC (r = -0.343; p < 0.05) and FRC (r = -0.394; p < 0.05) and positively to resting IC/TLC ratio (r = 0.512 ; p < 0.001).

**Table 1:** Demographic and baseline characteristics of COPD patients

	**Normal weight**	**Obese**
	**(n = 22)**	**(n = 22)**
Age (years)	66 ± 6	66 ± 6
Females/Males	9/13	7/15
BMI (kg/m^2^)	22 ± 2	34 ± 4**
FM (kg)	14.7 ± 5.9	36.2 ± 10.4**
FFM (kg)	46.2 ± 6.9	55.2 ± 8.8**
FFMI (kg/m^2^)	16.5 ± 1.7	19.8 ± 2.3**
MRC (0–4)	1 (0–4)	1 (0–4)
TLC (% pred)	128 ± 25	102 ± 22**
VC (% pred)	89 ± 17	77 ± 12**
FRC (% pred)	161 ± 34	137 ± 40*
RV (% pred)	187 ± 45	168 ± 56
FEV_1_ (% pred)	52 ± 13	50 ± 12
FEV_1_/VC (%)	50 ± 11	53 ± 9
FEF_50_/FIF_50_	0.32 ± 0.23	0.29 ± 0.12
TL_CO_ (% pred)	60 ± 16	78 ± 18**

*p value < 0.05.

**p value < 0.01.

Values are expressed as mean ± SD, median (range) or ratio.

**Figure 2:**
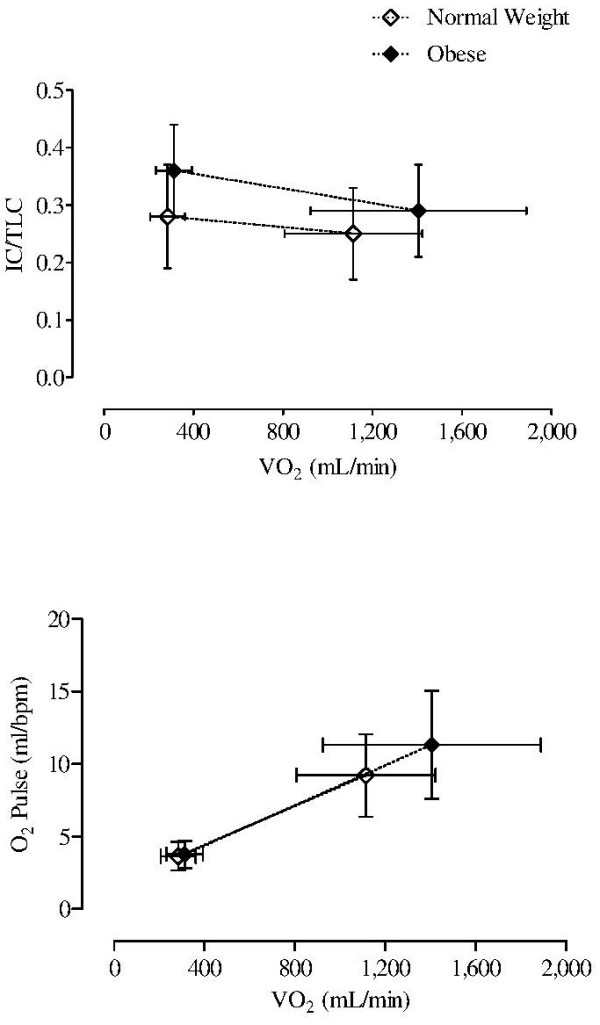
**Mean and standard deviation values of rest and peak IC/TLC ( *upper panel *) and O**_**2**_**Pulse ( *lower panel *) in relation to the corresponding rest and peak VO**_**2 **_**values in 22 normal weight and in 22 obese patients with COPD.** Mean IC/TLC value was significantly higher at rest (p < 0.01), but not at peak of exercise, mean O_2_Pulse and VO_2_ value were significantly higher at peak of exercise (p < 0.05), but not at rest of exercise in OB as compared to NW.

All the included patients completed the exercise test without any complications. The work rate increments during the exercise were not different between OB and NW patients (8.9 watts ± 2.9 vs 8.5 watts ± 2.5). When patients were asked to name the predominant symptom limiting the exercise test, 13 OB and 10 NW named exertion dyspnea, 6 OB and 8 NW named leg fatigue, whereas 3 OB and 4 NW named both symptoms. Exercise data are summarized in Table 2. The two groups of patients significantly differed in terms of peak VO_2_ when expressed as absolute value (p < 0.05) (Figure 2), but not when it was corrected by FFM and in terms of peak workload (p < 0.05). An AT was detected in 17 NW and in 19 OB patients and VO_2_ at AT was significantly higher in the OB group than in the NB group (1,145 ± 388 vs 821 ± 309 mL/min; p < 0.05). In addition, IC/TLC was significantly higher at rest (p < 0.01), but not at peak of exercise in OB as compared to NW (Figure 2), being > 0.25 at rest in 14 out of 22 (64%) NW and in 20 out of 22 (91%) OB patients (chi square = 4.659; p = 0.031) and > 0.25 at peak in 9 out of 22 (41%) NW and in 11 out of 22 (50%) OB patients (chi square = 0.0283; p = 0.866). The two groups of patients differed both in PETCO_2_ rest and in PETCO_2_ peak values, which were significantly higher in OB patients than in NW patients (p < 0.05), but not in PETCO_2_ peak-rest (8 ± 6 vs 6 ± 4 mm Hg; p = 0.218). As compared to NW, OB experienced lower leg fatigue (p < 0.05), but similar dyspnea on exertion.

**Table 2:** Exercise characteristics of 22 normal weight (NW) and 22 obese (OB) patients with COPD

	**Rest**		**Peak**	
	**NW**	**OB**	**NW**	**OB**
VO_2_ (mL/min)	284 ± 77	313 ± 81	1,114 ± 308	1,405 ± 482*
VO_2_/FFM (mL/kg)	6.3 ± 2.0	5.9 ± 1.9	24.1 ± 5.7	25.6 ± 7.6
Workload (watts)	0	0	74 ± 24	94 ± 36*
VE (L/min)	11.5 ± 2.2	10.7 ± 3.2	42.1 ± 13.1	40.8 ± 12.3
IC/TLC	0.28 ± 0.09	0.36 ± 0.08**	0.25 ± 0.08	0.29 ± 0.08
V_T_/VC	0.23 ± 0.08	0.27 ± 0.07	0.42 ± 0.15	0.55 ± 0.11**
V_T_/IC	0.36 ± 0.12	0.31 ± 0.09	0.73 ± 0.33	0.62 ± 0.11
PETCO_2_ (mm Hg)	30 ± 4	36 ± 3	36 ± 6	44 ± 7
SpO_2_ (%)	96 ± 2	95 ± 2	95 ± 2	94 ± 3
HR (bpm)	79 ± 14	84 ± 13	123 ± 18	124 ± 18
O_2_Pulse (mL/bpm)	3.6 ± 1.0	3.8 ± 0.9	9.2 ± 2.9	11.3 ± 3.7*
VAS dyspnea (mm/watt)	0	0	1.17 ± 1.0	1.12 ± 0.9
VAS fatigue (mm/watt)	0	0	1.19 ± 0.5	0.89 ± 0.4*

*p value < 0.05.

**p value < 0.01.

Values are expressed as mean ± SD.

The two groups of patients were not different in rest O_2_Pulse values, but they significantly differed in peak O_2_Pulse and in peak-rest O_2_Pulse values (7.6 ± 3 vs 5.6 ± 3 mL/bpm), which were both significantly higher in OB patients than in NW patients (p < 0.05) (Figure 2). In all patients, peak O_2_Pulse and peak-rest O_2_Pulse values were significantly and positively related to FFM (r = 0.496, p < 0.001 and r = 0.533, p < 0.001).

Lastly, in all patients significant positive correlations were found between peak workload and FFM (r = 0.584, p = 0.001) and IC/TLC at rest (r = 0.573, p = 0.001) and between VO_2_ and FFM (r = 0.552, p = 0.001) and IC/TLC at rest (r = 0.520, p = 0.001). Moreover, when all patients were categorized into two groups according to the median value of FFM (42 kg in females and 54 kg in males), patients with a FFM value higher than that of the median showed higher values in peak workload (99 watt ± 36 vs 74 watt ± 25, p < 0.05), peak VO_2_ (1438 ml/min ± 483 vs 1141 ml/min ± 342; p < 0.05) and in IC/TLC at rest (0.28 ± 0.09 vs 0.39 ± 0.07; p < 0.01). The regression equation generated by stepwise multiple regression analysis for peak workload and VO_2_, as dependent variables, included FFM and IC/TLC at rest. This model accounted for 43% and 37% of the total variance of the peak workload (in watts) and of VO_2_ (in mL) respectively, according to the following regression equations: W = -26.46 + 1.39 (FFM) + 126.85 (IC/TLC rest) and VO_2_ = -111.8 + 18.2 (FFM) + 1436.1 (IC/TLC rest).

## Discussion

The main finding of the present study is that obese patients with COPD, as compared to normal weight patients matched for age, gender and airflow obstruction, show greater fat-free mass and less lung hyperinflation at rest, as assessed by the IC/TLC ratio. When performing a maximal exercise test by cycling, obese patients compared to controls have greater exercise capacity both in terms of peak workload and in terms of oxygen uptake at peak, expressed as absolute value, but not when corrected for fat-free mass. In all patients, by means of multiple regression analysis fat-free mass and resting lung hyperinflation were found to be determining factors of the peak workload and oxygen uptake at peak. Lastly, as compared to controls obese patients experienced a comparable degree of dyspnoea and less leg fatigue on exertion.

Obesity may affect respiratory physiology both at rest and during exercise. Obese individuals show decreased respiratory system compliance, which results in a restrictive ventilatory defect. Low functional residual capacity and reductions in expiratory reserve volume increase the risk of expiratory flow limitation and airway closure during quiet breathing, especially in the supine position [28]. Moreover, in obese individuals, the increase of ventilation during exercise can further induce gas trapping and a rise of EELV [29]. Paradoxically, the dynamic increase in EELV may result in a mechanical advantage in obese subjects while exercising, since tidal volume shifts to the more compliant portion of the pressure-volume relation of the respiratory system [6]. The reduction in operating lung volumes during exercise may have favourable influences especially in obese patients with COPD, in whom obesity may counterbalance lung hyperinflation, thereby significantly improving their exercise tolerance [7,8].

In the present study, as compared to normal weight patients with COPD, the COPD patients with combined obesity developed during exercise, as assessed by the IC/TLC ratio at peak of exercise, dynamic hyperinflation of comparable magnitude and a similar degree of ventilatory limitation, as assessed by the difference in peak-rest PETCO_2_ value. Despite this, obese patients showed greater maximal exercise capacity both in terms of peak workload and in terms of oxygen uptake, thanks to their better starting conditions consisting in less lung hyperinflation at rest. Our results confirm and extend those of previous reports [7,8]. In this study, we also provided the first evidence that obese COPD patients, compared to normal weight COPD patients had a greater amount of lean body mass, which together with lung hyperinflation was a determinant factor of maximal exercise capacity in all patients. Therefore, both pulmonary and non-pulmonary factors may explain the preservation of exercise tolerance in patients with combined COPD and obesity.

It is not surprising to find a greater amount of fat-free mass in obese patients in comparison to normal weight controls, since the increase in fat-free mass represents an adaptation to the sustained loading effect from excessive adipose tissue. Previously, Babb et al. [13] by investigating the relationship between EELV, body composition and fat distribution in a group of lean and obese young adult men and women, incidentally reported that the lean mass was increased in obese subjects by 10 kg, both in men and in women. Similarly, Wood et al. [14] studying the aerobic power in overweight and obese young adults, reported a significant increase of fat-free mass in obese subjects. The greater amount of lean body mass in obese COPD patients could be an explanation of the seemingly counterintuitive paradox of obesity, which from a risk factor results in a survival advantage of chronic disease states or conditions associated with wasting disease at the population level [5].

Lean body mass may have favourable effects on exercise tolerance in patients with wasting diseases, such as COPD [30]. Notably, the skeletal muscle pump has local and systemic circulatory effects, since it may enhance venous return, central venous pressure, end-diastolic volume, and thus stroke volume and cardiac output, by expulsing the peripheral venous blood volume during exercise [31]. It is of note that in this study we found that in comparison to controls, obese patients showed a better cardiovascular response to exercise, expressed as oxygen pulse, which can be considered as a reliable surrogate marker of the stroke volume [32]. In addition, oxygen pulse was strictly related to FFM in all COPD patients.

This study has some limitations. Firstly, our obese patients showed mild-to-moderate obesity and our results cannot be generalizable to all obese patients, including those with morbid obesity. Secondly, in our study patients experienced maximal exercise capacity by using cycle ergometry, therefore, our results cannot be applicable to other forms of exercise, such as running on a treadmill, where the metabolic load is greater. Thirdly, in our patients body composition was assessed by bioelectrical impedance analysis, which is sensitive to the hydration status of the subject whereas, distribution of body fat was not analysed. However, our patients showed normal hydration status, as assessed by means of urinalysis, including urine specific gravity. Moreover, in both men and women with obesity the decrease in end-expiratory lung volume appears to be related to the cumulative effect of increased chest wall fat rather than to any specific regional chest wall fat distribution [13]. Lastly, our study is a cross-sectional study, therefore, on the basis of our results we can only infer and not establish the contributors of exercise tolerance in obese patients with COPD. Thus, a further longitudinal study on exercise tolerance in obese patients, who change their weight and body composition over time, is needed.

## Conclusions

OB with COPD, as compared to NW patients matched for age, gender and airflow obstruction, had greater FFM and less resting lung hyperinflation and showed greater maximal exercise capacity. Pulmonary and non-pulmonary factors may explain the preservation of exercise tolerance in patients with COPD associated with obesity.

## Abbreviations

AT: Anaerobic threshold; BMI: Body mass index; COPD: Chronic obstructive pulmonary disease; CPET: Cardiopulmonary exercise test; FEF_50_: Forced expiratory flow measured at 50% of FVC; FEV_1_: Forced expiratory volume in the 1^st^ second; FIF_50_: Forced inspiratory flow measured at 50% of FVC.; FFM: Fat-free mass; FFMI: Fat-free mass index; FM: Fat mass; FRC: Functional residual capacity; FVC: Forced vital capacity; HR: Heart rate; IC: Inspiratory capacity; O_2_Pulse: Oxygen pulse; RV: Residual volume; SD: Standard deviation; TLC: Total lung capacity; TLco: Lung diffusion capacity for carbon monoxide; VAS: Visual analogue scale; VC: Vital capacity; VCO_2_: Carbon dioxide production; VE: Minute ventilation; VO_2_: Oxygen uptake; V_T_: Tidal volume.

## Competing interests

The authors declare that they have no competing interests.

## Authors’ contributions

MA served as the primary author. She developed the study protocol, participated in the patient recruitment and statistical analysis and drafted the manuscript and she is the guarantor of the entire manuscript. PT, SR, ET and MRG participated in the design of the study and helped with patient recruitment. EM participated in the coordination of the study. AC developed the study protocol, interpreted study data, contributed to and reviewed drafts of the manuscript. All authors read and approved the final manuscript.

## Pre-publication history

The pre-publication history for this paper can be accessed here:

http://www.biomedcentral.com/1471-2466/14/96/prepub

## References

[B1] Adams KF Schatzkin A Harris TB Kipnis V Mouw T Ballard-Barbash R Hollenbeck A Leitzmann MF Overweight, obesity, and mortality in a large prospective cohort of persons 50 to 71 years old N Engl J Med 2006 355 763 778 10.1056/NEJMoa055643 16926275

[B2] Franssen FM O’Donnell DE Goossens GH Blaak EE Schols AM Obesity and the lung: 5. Obesity and COPD Thorax 2008 63 1110 1117 10.1136/thx.2007.086827 19020276

[B3] Wilson DO Rogers RM Wright EC Anthonisen NR Body weight in chronic obstructive pulmonary disease. The national institutes of health intermittent positive-pressure breathing trial Am Rev Respir Dis 1989 139 1435 1438 10.1164/ajrccm/139.6.1435 2658702

[B4] Landbo C Prescott E Lange P Vestbo J Almdal TP Prognostic value of nutritional status in chronic obstructive pulmonary disease Am J Respir Crit Care Med 1999 160 1856 1861 10.1164/ajrccm.160.6.9902115 10588597

[B5] Kalantar-Zadeh K Horwich TB Oreopoulos A Kovesdy CP Younessi H Anker SD Morley JE Risk factor paradox in wasting diseases Curr Opin Clin Nutr Metab Care 2007 10 433 442 10.1097/MCO.0b013e3281a30594 17563461

[B6] O’Donnell DE O’Donnell CD Webb KA Guenette JA Respiratory consequences of mild-to-moderate obesity: impact on exercise performance in health and in chronic obstructive pulmonary disease Pulm Med 2012 2012 818925 23097698 10.1155/2012/818925 PMC3477561

[B7] Ora J Laveneziana P Ofir D Deesomchok A Webb KA O'Donnell DE Combined effects of obesity and chronic obstructive pulmonary disease on dyspnea and exercise tolerance Am J Respir Crit Care Med 2009 180 964 971 10.1164/rccm.200904-0530OC 19897773

[B8] Ora J Laveneziana P Wadell K Preston M Webb KA O'Donnell DE Effect of obesity on respiratory mechanics during rest and exercise in COPD J Appl Physiol 2011 111 10 19 10.1152/japplphysiol.01131.2010 21350021

[B9] Kim HC Mofarrahi M Hussain SNA Skeletal muscle dysfunction in patients with chronic obstructive pulmonary disease Int J Chron Obstruct Pulmon Dis 2008 3 637 658 19281080 10.2147/copd.s4480 PMC2650609

[B10] Baarends EM Schols AMWJ Mostert R Wouters EFM Peak exercise response in relation to tissue depletion in patients with chronic obstructive pulmonary disease Eur Respir J 1997 10 2807 2813 10.1183/09031936.97.10122807 9493665

[B11] Schols AM Mostert R Soeters PB Wouters EF Body composition and exercise performance in patients with chronic obstructive pulmonary disease Thorax 1991 46 695 699 10.1136/thx.46.10.695 1750015 PMC463385

[B12] Teopompi E Tzani P Aiello M Ramponi S Andrani F Marangio E Clini E Chetta A Fat free mass depletion is associated to poor exercise capacity irrespective of dynamic hyperinflation in COPD patients Respir Care 2014 59 718 725 10.4187/respcare.02709 24170915

[B13] Babb TG Wyrick BL DeLorey DS Chase PJ Feng MY Fat distribution and end-expiratory lung volume in lean and obese men and women Chest 2008 134 704 711 10.1378/chest.07-1728 18641101

[B14] Wood RE Hills AP Hunter GR King NA Byrne NM VO_2max_ in overweight and obese adults: do they meet the threshold criteria? Med Sci Sports Exerc 2010 423 470 477 19952821 10.1249/MSS.0b013e3181b666ad

[B15] Pauwels RA Buist AS Calverley PM Jenkins CR Hurd SS GOLD Scientific Committee Global strategy for the diagnosis, management, and prevention of chronic obstructive pulmonary disease. NHLBI/WHO global initiative for chronic obstructive lung disease (GOLD) workshop summary Am J Respir Crit Care Med 2001 163 1256 1276 10.1164/ajrccm.163.5.2101039 11316667

[B16] Miller MR Hankinson J Brusasco V Burgos F Casaburi R Coates A Crapo R Enright P Van der Grinten CP Gustafsson P Jensen R Johnson DC MacIntyre N McKay R Navajas D Pedersen OF Pellegrino R Viegi G Wanger J ATS/ERS Task Force Standardisation of spirometry Eur Respir J 2005 26 319 338 10.1183/09031936.05.00034805 16055882

[B17] Wanger J Clausen JL Coates A Pedersen OF Brusasco V Burgos F Casaburi R Crapo R Enright P Van der Grinten CP Gustafsson P Hankinson J Jensen R Johnson D Macintyre N McKay R Miller MR Navajas D Pellegrino R Viegi G Standardisation of the measurement of lung volumes Eur Respir J 2005 26 511 522 10.1183/09031936.05.00035005 16135736

[B18] Quanjer PH Tammeling GJ Cotes JE Pedersen OF Peslin R Yernault JC Lung volumes and forced ventilatory flows. Report working party standardization of lung function tests, European community for steel and coal. official statement of the European respiratory society Eur Respir J 1993 6 Suppl 5 40 8499054

[B19] Cotes JE Chinn DJ Quanjer PH Roca J Yernault JC Standardization of the measurement of transfer factor (diffusing capacity). Report working party standardization of lung function tests, European community for steel and coal. official statement of the European respiratory society Eur Respir J 1993 6 Suppl 41 52 8499053

[B20] ATS/ACCP Statement on cardiopulmonary exercise testing Am J Respir Crit Care Med 2003 167 211 277 12524257 10.1164/rccm.167.2.211

[B21] Stubbing DG Pengelly LD Morse JL Jones NL Pulmonary mechanics during exercise in subjects with chronic airflow obstruction J Appl Physiol 1980 49 511 515 7204175 10.1152/jappl.1980.49.3.511

[B22] Brooks SM Surveillance for respiratory hazards ATS News 1982 8 12 16

[B23] Tzani P Piepoli MF Longo F Aiello M Serra W Maurizio AR Olivieri D Chetta A Resting lung function in the assessment of the exercise capacity in patients with chronic heart failure Am J Med Sci 2010 339 210 215 10.1097/MAJ.0b013e3181c78540 20220330

[B24] Segal KR Van Loan M Fitzgerald PI Hogdon JA Van Itallie TB Lean body mass estimation by bioelectrical impedance analysis: a four-site cross-validation study Am J Clin Nutr 1988 47 7 14 3337041 10.1093/ajcn/47.1.7

[B25] Kopelman PG Obesity as a medical problem Nature 2000 404 635 643 10766250 10.1038/35007508

[B26] Casanova C Cote C De Torres JP Aguirre-Jaime A Marin JM Pinto-Plata V Celli BR Inspiratory-to-total lung capacity ratio predicts mortality in patients with chronic obstructive pulmonary disease Am J Respir Crit Care Med 2005 171 591 597 10.1164/rccm.200407-867OC 15591470

[B27] Tzani P Aiello M Elia D Boracchia L Marangio E Olivieri D Clini E Chetta A Dynamic Hyperinflation is Associated with a Poor Cardiovascular Response to Exercise in COPD Patients Respir Res 2011 12 150 10.1186/1465-9921-12-150 22074289 PMC3225311

[B28] Lin C-K Lin C-C Work of breathing and respiratory drive in obesity Respirology 2012 17 402 411 10.1111/j.1440-1843.2011.02124.x 22212441

[B29] Ofir D Laveneziana P Webb KA O'Donnell DE Ventilatory and perceptual responses to cycle exercise in obese women J Appl Physiol 2007 102 2217 2226 10.1152/japplphysiol.00898.2006 17234804

[B30] Visca D Aiello M Chetta A Cardiovascular function in pulmonary emphysema Biomed Res Int 2013 2013 184678 24369007 10.1155/2013/184678 PMC3866814

[B31] Sheriff D Point: the muscle pump raises muscle blood flow during locomotion J Appl Physiol 2005 99 371 372 10.1152/japplphysiol.00381.2005 16036908

[B32] Whipp BJ Higgenbotham MB Cobb FC Estimating exercise stroke volume from asymptotic oxygen pulse in humans J Appl Physiol 1996 81 2674 2679 9018521 10.1152/jappl.1996.81.6.2674

